# Characteristics of miRNAs Present in Bovine Sperm and Associations With Differences in Fertility

**DOI:** 10.3389/fendo.2022.874371

**Published:** 2022-05-19

**Authors:** Nicholas Werry, Stewart J. Russell, Daniel J. Gillis, Sarah Miller, Katie Hickey, Steven Larmer, Michael Lohuis, Clifford Librach, Jonathan LaMarre

**Affiliations:** ^1^Department of Biomedical Sciences, The University of Guelph, Guelph, ON, Canada; ^2^CReATe Fertility Centre, Toronto, ON, Canada; ^3^School of Computer Science, The University of Guelph, Guelph, ON, Canada; ^4^Semex Alliance, Guelph, ON, Canada; ^5^Department of Obstetrics and Gynecology, University of Toronto, Toronto, ON, Canada; ^6^Department of Physiology, University of Toronto, Toronto, ON, Canada; ^7^Institute of Medical Sciences, University of Toronto, Toronto, ON, Canada

**Keywords:** miRNAs, sperm, fertility, bovine, spermatogenesis, embryo, pathway analysis

## Abstract

Small non-coding RNAs have been linked to different phenotypes in bovine sperm, however attempts to identify sperm-borne molecular biomarkers of male fertility have thus far failed to identify a robust profile of expressed miRNAs related to fertility. We hypothesized that some differences in bull fertility may be reflected in the levels of different miRNAs in sperm. To explore such differences in fertility that are not due to differences in visible metrics of sperm quality, we employed Next Generation Sequencing to compare the miRNA populations in *Bos taurus* sperm from bulls with comparable motility and morphology but varying Sire Conception Rates. We identified the most abundant miRNAs in both populations (miRs -34b-3p; -100-5p; -191-5p; -30d-4p; -21-5p) and evaluated differences in the overall levels and specific patterns of isomiR expression. We also explored correlations between specific pairs of miRNAs in each population and identified 10 distinct pairs of miRNAs that were positively correlated in bulls with higher fertility and negatively correlated in comparatively less fertile individuals. Furthermore, 8 additional miRNA pairs demonstrated the opposite trend; negatively correlated in high fertility animals and positively correlated in less fertile bulls. Finally, we performed pathway analysis to identify potential roles of miRNAs present in bull sperm in the regulation of specific genes that impact spermatogenesis and embryo development. Together, these results present a comprehensive picture of the bovine sperm miRNAome that suggests multiple potential roles in fertility.

## 1 Introduction

The frequency of successful pregnancy per round of artificial insemination (AI) is approximately 38% in Ontario cattle (1). Improved fertility and conception yields significant economic advantages for producers and is therefore a significant focus of breeding strategies and a key criterion when selecting bulls for AI (2, 3). Currently, one of the major approaches to male fertility assessment is Computer Assisted Semen Analysis (CASA), which typically predicts approximately 50–60% of the variability in fertility (4, 5). However, these measurements are often inconsistent with manual semen analysis results (6) and fail to reflect all factors influencing fertilization success, as measured by Sire Conception Rate (SCR). SCR is a key metric of fertility performance. By way of example, semen from a bull with an SCR of +2.0 yields a pregnancy rate 2% higher than the herd average in contrast to an SCR of -2.0 which indicates a 2% lower than average pregnancy rate.

Several researchers have investigated biochemical methods of evaluating semen to better predict SCR. Studies have surveyed seminal protein composition (7–9), single nucleotide genetic polymorphisms (10, 11), and the expression of small noncoding RNAs (sncRNAs) (12, 13). Typically, sncRNA-based investigations seek to identify differentially expressed sequences that reflect fertility (14–18). Many have been identified in bovine semen and transcripts present in other species’ semen have been implicated in spermatogenesis (14), fertility (15), and embryogenesis (16, 17). However, investigations have yet to identify a robust biochemical approach capable of distinguishing idiopathic variations in fertility.

miRNAs are a specific class of sncRNA that are implicated in many diverse traits from reproductive development to cancer biology (19–21). Mature miRNAs are the result of a processing pathway beginning with larger pri-miRNAs that are transcribed from the genome. These are cleaved in the nucleus by Drosha and DGCR8 to form pre-miRNAs, which are exported to the cytoplasm, and further cleaved by Dicer to yield mature miRNAs approximately 20 nucleotides in length (22). Mature miRNA sequences associate with Argonaute proteins which are targeted to complementary sequences in mRNAs that are usually found in the 3’UTR. Targeted binding supresses gene expression through a multi-step process involving translation inhibition, deadenylation, and target degradation (23). Due to their stability and multiple potential roles in biological processes, miRNAs are often employed as biomarkers. They have been extensively evaluated in the fields of reproduction and fertility (12, 18–20, 24) and have been recently reviewed elsewhere (25).

Sperm-borne miRNAs may originate from several different sources. miRNAs are implicated in virtually every stage of spermatogenesis, from differentiation of male germline stem cells (26, 27) to morphological changes in spermiogenesis (14). Their presence in mature sperm may be residual from regulatory events during spermatogenesis, or they may have been acquired through fusion with epididysomes during post-testicular maturation (28). Epididymis-derived miRNAs appear to be vital for proper sperm development, and alterations in their delivery to sperm during epididymal transit is linked to reduced motility (29). Regardless of their origin, miRNAs in spermatozoa may have functional roles outside the sperm cell itself, as RNAs are delivered to the zygote along with the paternal genome (30), and have been reported to influence early embryonic development (31, 32).

Overall, miRNAs present in sperm likely reflect specific characteristics (and the overall success) of regulatory events in “upstream” processes such as spermatogenesis (14, 26, 27) and epididymal transit (28, 29). Subsequently, they are likely to participate in “downstream” post-fertilization events such as fertilization and cleavage (31, 32).

In attempts to understand their specific roles in fertility, several researchers have attempted to identify sperm-borne miRNAs differentially expressed between sperm with high or low *in vitro* blastocyst rates (12) and *in vivo* fertility (18). Interestingly, differentially expressed transcripts identified by those studies do not appear to overlap. However, both have relatively small sample sizes. To date, a robust and consistent list of miRNAs correlated with fertility has yet to be ascertained, likely due to the complex network of molecular interactions in which each miRNA may participate. Fertility is a highly complex polygenic trait with over 2700 implicated genes (33). The epistatic, synthetic, and suppressive interactions between these genes at a gene-gene level are further complicated by possible epistasis between miRNAs and genes (34), and miRNA-miRNA interactions (35, 36).

Specific miRNAs often have sequence variants, known as isomiRs, that are produced from the same locus but differentially processed to yield alternative sequences (37). IsomiRs sharing the same seed sequence were once thought to have identical gene targeting properties, but recent evidence has suggested that complementarity outside of the seed sequence likely adds complexity to these interactions (38). Therefore, simple analysis of differential miRNA expression is not likely to yield a complete picture of the dynamic roles that sperm-borne miRNAs play and more complex comparisons are likely necessary.

Previous research in humans has identified miRNA pairs with correlated expression in semen of fertile males, while these same correlations are absent in subfertile individuals (39). These correlations arise from various relationships between miRNAs. For example, miR-34b/c and miR-449a/b/c are known to be functionally redundant within sperm, thus the expression of both clusters is linked to the same phenotypic outcomes (14, 40). In the present study, we examined differential expression and isomiR differences between sperm samples from higher and lower fertility bulls. and extended previous correlation-based approaches to examine differentially correlated miRNA-miRNA relationships; in an attempt to more fully describe transcriptomic elements underlying small, but significant, variation in male bovine fertility.

## 2 Materials and Methods

### 2.1 Bull Selection

Bos taurus (Holstein) semen was provided through a collaboration with Semex (Guelph, Ontario, Canada) and separated into two groups based on SCR: high fertility (HF) bulls with SCR ≥ 1.0 (n = 7, mean: 2.1 ± 0.7), and low fertility (LF) with SCR ≤ –1.0 (n = 6, mean: –2.7 ± 1.1). All samples were of comparable age and exceeded acceptable parameters for sperm quality; no significant differences were present between groups with the exception of SCR (**Table 1**).

**Table 1:** Motility metrics show no significant difference between high and low fertility bulls.

Metric	High	Low	p-value
Sire conception rate	2.01 ± 0.25	-2.73 ± 0.40	*1.12e-05
Age at collection (days)	967 ± 264	898 ± 102	0.80
Total motility 0h post-thaw (%)	62.48 ± 2.59	57.72 ± 1.97	0.21
Progressive motility 0h post-thaw (%)	32.15 ± 3.12	28.68 ± 1.49	0.38
Total motility 2h post-thaw (%)	56.87 ± 2.08	50.04 ± 2.75	0.10
Progressive motility 2h post-thaw (%)	27.41 ± 1.59	22.02 ± 1.87	0.07
Curvilinear velocity (VCL) (µm/s)	103.79 ± 3.55	104.55 ± 2.42	0.87
Average path velocity (VAP) (µm/s)	84.1 ± 3.25	78.71 ± 3.07	0.29
Straight line velocity (VSL) (µm/s)	76.57 ± 2.83	71.77 ± 2.78	0.29
Linear coefficient (LIN) (%)	73.68 ± 2.49	68.73 ± 1.53	0.15
Straightness coefficient (STR) (%)	89.46 ± 1.04	90.81 ± 0.86	0.37
Wobble coefficient (WOB) (%)	81.57 ± 2.42	75.36 ± 1.6	0.08
Lateral head displacement (ALH) (µm)	3.31 ± 0.25	3.63 ± 0.12	0.32
Beat cross efficiency (BCF) (Hz)	12.37 ± 0.36	12.53 ± 0.42	0.80

Data is presented as mean ± SEM. p-values were calculated by 2 tailed unpaired t-test, no categories show significant difference below a threshold of 0.05. * indicates significant result.

### 2.2 Sperm Isolation

Motile sperm from 500 µL of semen was separated by Percoll gradient as previously described (19). Briefly, 500 µL of cryopreserved semen was loaded onto a 2 mL Percoll gradient, consisting of a 45% Percoll solution layered atop an equal volume of 90% Percoll. After centrifugation at 1800 rpm for 30 minutes, the bottom 1.5 mL (consisting of highly motile sperm) was transferred to 5 mL of sperm HEPES/TALP and centrifuged for 10 minutes at 400 x g. The bottom 1.5 mL of the wash was then transferred to a 1.5 mL tube and centrifuged for 5 minutes at 700 x g at 4°C. Supernatant was discarded, samples were flash frozen in liquid nitrogen and stored at -80°C.

### 2.3 RNA Isolation

Total RNA was extracted using Macherey-Nagel NucleoSpin miRNA extraction kit. Sperm pellets were suspended in 300 µL Buffer ML, sonicated on ice for 20 seconds at setting 15 on a MICROSON XL 2000 Ultrasonic Liquid Processor, then incubated at room temperature for 15 minutes. Extraction continued according to the manufacturer’s protocol, eluting in 100 µL of 95°C nuclease-free water and immediate re-elution. Samples were concentrated by centrifugal evaporation to 20 µL and stored at -80°C.

### 2.4 Library Preparation and Illumina Sequencing

Small RNA libraries were prepared using NEXTflex Small RNA-Seq Kit v3 (PerkinElmer) according to the manufacturer’s instructions with the following approaches: adapters diluted 1:4, 3’ ligation performed for 18h, samples amplified for 23 PCR cycles, then gel-free size selected according to optional step H1 in the instructions. Libraries were size selected to 140–170 bp using the SAGE Pippin Prep system and repurified by gel-free size selection H1. Sequencing of 1 x 75 bp was performed with an Illumina NextSeq high output kit, resulting in 81,250,738 total reads.

### 2.5 Bioinformatic Analysis

#### 2.5.1 Sequence Annotation

Small non-coding RNA sequencing data processing and analysis was performed as described previously (41). Briefly, raw sequence QC and processing was performed with the FASTX Toolkit (42) and command line tools available at http://www.smallrnagroup.uni-mainz.de/software.html. Reads were filtered for lengths above 18 nt and Phred scores above 30. Small RNAs were annotated with Unitas v1.7.0 using the latest available public small RNA databases to annotate input sequences (43) and aligned to small RNA databases (44–48).

#### 2.5.2 Differential Expression Analyses

Per-sample normalized read counts were collapsed by annotation, then filtered for miRNAs. The remaining sequences were analyzed using the DEseq2 pipeline for differential expression (49).

For isomiR differential expression analysis, non-collapsed data were used to perform unpaired 2-tailed t-tests between fertility conditions for each isomiR sequence. Benjamini-Hochberg correction was performed with respect to the number of isomiRs contributing to each miRNA annotation, an intentionally permissive threshold as the analysis was exploratory in nature.

#### 2.5.3 Correlational Analysis

Normalized miRNA expression levels, as described above, were analyzed in R [Version 4.0.2 (50)] by first extracting all records where expression levels (eg. normalized read counts) for specific miRNAs were known. miRNAs with mean read counts < 100, or lacking reads in two or more samples were excluded, yielding 247 uniquely annotated pairs. The relative levels of any two miRNAs (nominally miRNA-A, and miRNA-B) were then examined, resulting in 30381 unique two-way comparisons. Two correlation coefficients were then determined: the first considering the relationship between miRNA-A and miRNA-B expression levels within the high fertility group, and the second considering the relationship between miRNA-A and miRNA-B expression within the low fertility group. Comparisons with a statistically significant (p<0.05) Spearman correlation coefficient for both fertility groups independently were retained, leading to 140 comparisons. For each comparison a linear model was created: miRNA(A) = beta0 + beta1 ∗ miRNA − B + beta2 ∗ LOW  + beta3 ∗ LOW ∗ miRNA   B , where miRNA(A) and miRNA(B) represent expression of miRNA(A) and miRNA(B), respectively. Betas represent the parameters of the linear model, and LOW is an indicator variable which is set to 1 if the gene expression values are specific to the low fertility group, and 0 if the gene expression values are specific to the high fertility group. Parameter estimates for beta0, beta1, beta2, and beta3 and their associated p-values were extracted. Significant values for beta3 would indicate a significant difference in the slopes, representing a difference in the levels of candidate miRNAs in enhanced and lower fertility bulls. Of the 140 linear models explored, the 23 with statistically significant estimates for beta1 and beta3 (and therefore showing different slopes) were retained. To control the false discovery rate, the Benjamini-Hochberg procedure was used, yielding 18 statistically significant comparisons (51).

### 2.6 Gene Set Enrichment Analysis

Given the limited availability of interaction data for bovine miRNA sequences, miRNAs were converted to their human homologs for gene set enrichment and targeting analysis (**Supplementary Table S1**). DIANA-miRPath v3.0 was used to identify gene union results from Tarbase v7.0, with p < 0.05. The 20 most significant terms were plotted for KEGG and each GO category.

For gene targeting analysis, a more comprehensive search was performed to identify miRNA-gene interactions from several databases of validated interactions: miRecords, miRTarBase, and TarBase. When investigating correlated pairs, resulting gene targets were filtered to select only those shared by both miRNAs. Results were further filtered to identify targets with documented expression in relevant cellular contexts: mature testis by cross-referencing with findings from Gao et al. (52), or early embryos by cross referencing the EmbryoGENE database for expression at the GV, MII, 1-cell, 2-cells, 4-cells, 8-cells-early, or 8-cells-late stage (53).

## 3 Results

### 3.1 Small Non-Coding RNA

**Figure 1:**
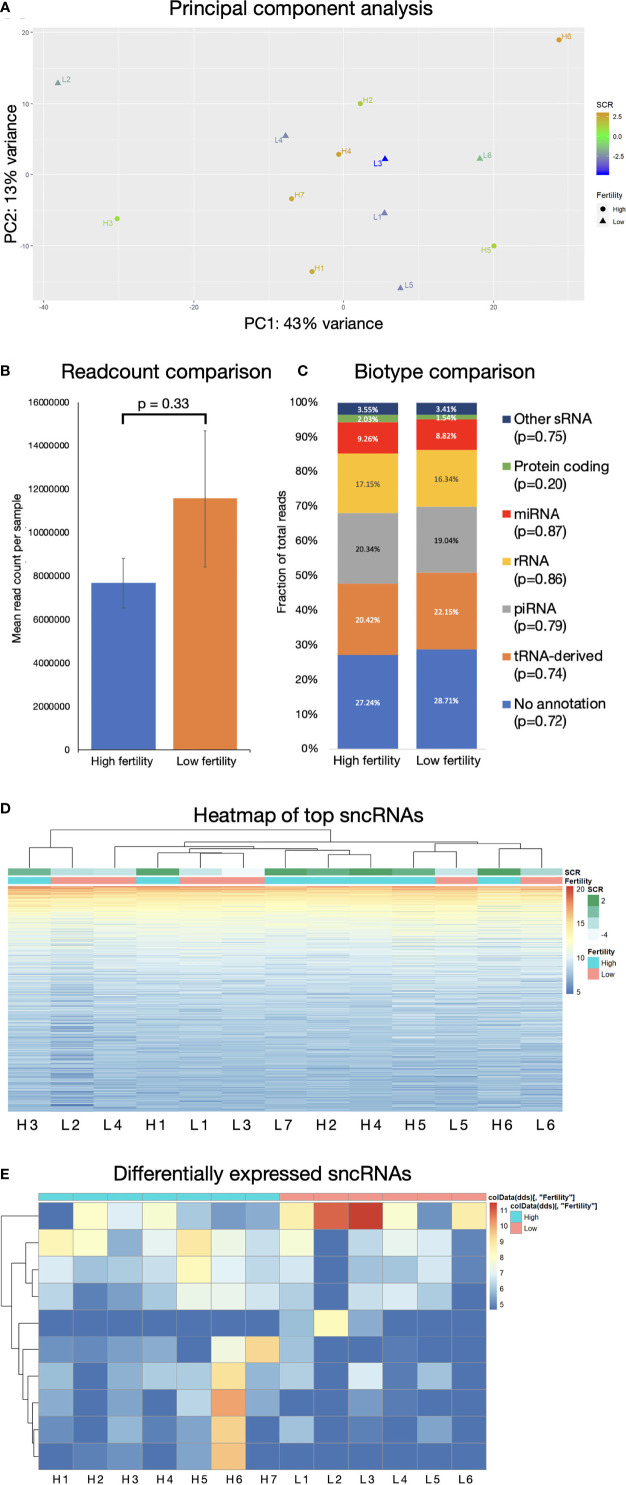
sncRNA populations are broadly similar across fertility groups. **(A)** Principal component analysis does not show significant clustering correlated with fertility. Mean read count/sample **(B)** and biotype composition **(C)** show no significant difference by 2-tailed T-test, error bars are ± SD. **(D)** Hierarchical clustering of 1000 most expressed sncRNAs does not associate by fertility conditions. **(E)** Significantly differentially expressed sncRNAs below significance threshold of FDR ≤ 0.05.

81,250,738 reads were generated by Illumina NextSeq analysis of 13 samples: 6 “low fertility” with SCR ≤ –1 and 7 “high fertility” with SCR ≥ +2. (**Figure 1A**). Fertility groups do not form independent clusters when sncRNA populations are explored by principal component analysis (**Figure 1B**). The most abundant biotypes by relative proportion of annotated sequences were tRNAs (33.5%), piRNAs (27.3%), miRNAs (18.2), rRNAs (14.2%), miscellaneous RNAs (3.0%), mRNAs, (2.1%), lncRNAs (1.1%), and other biotypes comprising less than 1% of expressed sncRNAs (ribozymes, snRNAs, snoRNAs, scaRNAs, lincRNAs). Abundance of all biotypes is consistent across fertility groups, FDR = 0.891 (**Figure 1C**). Unsupervised hierarchical clustering of the most abundant individual sequences does not group samples by their fertility condition (**Figure 1D**). This suggests that the population of sncRNA sequences in bovine sperm does not exhibit large-scale systemic variation related to idiopathic fertility variation.

Some individual sncRNA sequences displayed statistically significant differential expression between fertility conditions, (**Figure 1E**), however the majority of these were of very low abundance, and any statistical significance is likely driven by high expression in a limited number of animals. Filtering to remove sequences with fewer than 100 reads revealed no significantly differentially expressed non-miRNA sncRNAs.

### 3.2 miRNAs

#### 3.2.1 Population Characteristics

**Figure 2:**
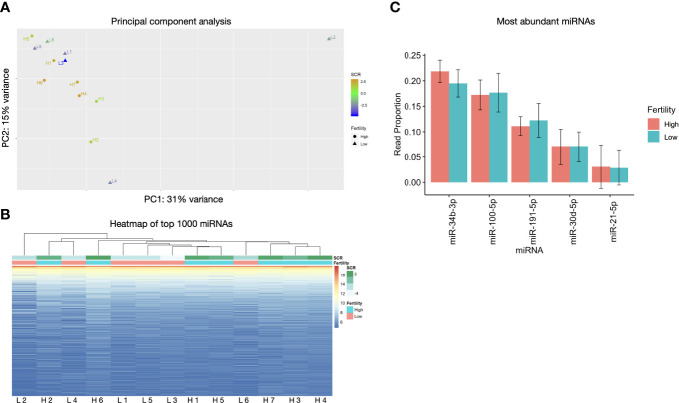
miRNA populations are broadly similar across fertility groups. **(A)** Principal component analysis does not show significant clustering correlated with fertility. **(B)** Heatmap of top 1000 expressed miRNAs shows minimal association between samples of like fertility conditions. **(C)** The 5 most abundant miRNAs exhibit no significant differences and are ranked the same in enhanced and standard fertility animals. Error bars are ± SD.

Sperm miRNA profiles did not show significant differences between low and high fertility animals. Principal component analysis revealed no clustering with respect to fertility and hierarchical clustering displays showed only minimal association of some sequences overexpressed in high fertility animals (**Figures 2A, B**). Sequence annotation revealed 1431 distinct miRNAs. Of these, the most abundant 5 contributed 60% of the total miRNA reads. Expression of these abundant sequences is consistent between bulls with high or low fertility (**Figure 2C**). Full annotated read counts are available in **Supplementary Table S2**.

Of the 1431 miRNAs, only 5 sequences with very low read counts exhibited differential expression, with an FDR ≤ 0.05 (**Table 2**). While the statistical analysis supports unequivocal differences between these miRNAs in the two groups, 3 of these miRNAs are absent in the low fertility group and present at very low read counts in the high group, which led us to eliminate these 3 from further analysis. The results are included here for completeness, but based on the low read counts, we cannot implicate them in the fertility differences under study.

**Table 2:** Differentially expressed miRNAs.

miRNA	High Mean	Low Mean	Log2 FC	SE	FDR
bta-miR-2450c-3p	5.53	0	–30.00	3.45	4.441E-15
bta-miR-2311-5p	4.09	0	–30.00	3.54	1.59E-14
ppc-mir-2274-5p	1.61	0	–30.00	3.56	1.59E-14
bta-miR-409a-3p	60.32	23.70	–3.16	0.77	0.0137
bta-miR-543	135.97	51.85	–3.32	0.83	0.0177

Log2 fold differences are maximum-likelihood estimates calculated with respect to high/low, sequences shown have FDR ≤ 0.05.

#### 3.2.2 IsomiR Composition

**Figure 3:**
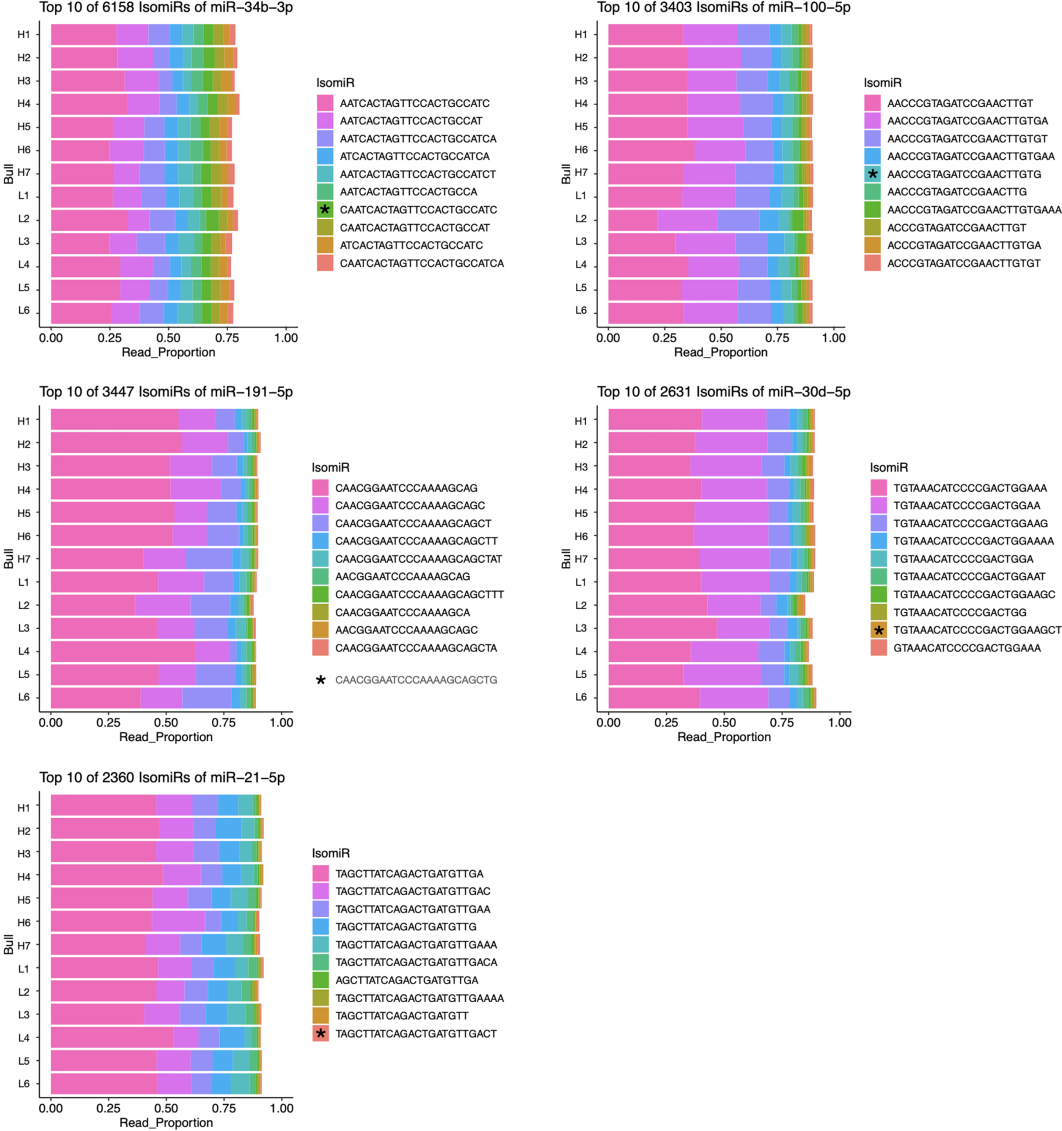
Most abundant isomiRs of five most abundant miRNAs in bovine sperm. IsomiRs ranked by proportion of reads contributed by each sequence for given miRNA. The relative abundance of miRBase consensus sequences (indicated by *) are: miR-34b-3p, 4.13%; miR-100-5p, 4.53%; miR-191-5p, 0.41%; miR-30d-5p, 0.91%; miR-21-5p, 0.44%.

IsomiRs are minor variation in sequences of a given miRNA that are associated with differences in processing and potential targeting as described above. Investigating the sequences of expressed miRNAs revealed that high isomiR diversity exists in bovine sperm, and a small subset of isomiRs is often responsible for the majority of a given miRNA’s reads. Interestingly, the most abundant isomiR sequence was often different from the consensus sequence found in miRBase (46). Of the five most abundant miRNAs in sperm, no miRBase sequence represented more than 5% of the total reads (46). The most abundant isomiRs of these abundant miRNAs (miR-34b-3p, miR-100-5p, miR-191-5p, miR-30d-4p, and miR-21-5p) are presented in **Figure 3**. No individual isomiR was found to have significant (FDR <0.05) differential expression between fertility conditions.

#### 3.2.3 Differential Correlation

**Figure 4:**
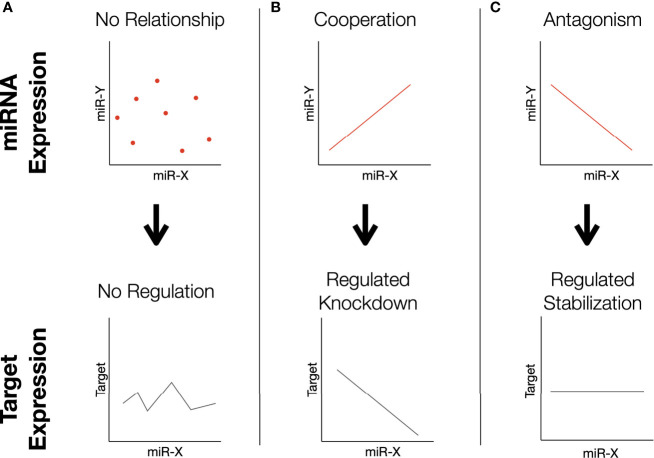
Proposed dynamics of miRNA correlations. In this correlation-based analysis, we assume miRNAs influence the same direct phenotype related to some aspect of fertility. Note that this mechanism may manifest by the correlated pairs targeting the same gene (as depicted), or different aspects of a functional pathway. **(A)** Two miRNAs whose expression is not correlated may result in variable expression of the shared target. **(B)** Cooperating miRNAs effectively regulate knockdown: low levels of both permits high expression, high levels of both silence it. **(C)** Antagonistic miRNAs have inverse expression patterns; high levels of one are associated with low levels of the other. The relationship ensures moderated expression of the target, as the overall abundance of both miRNAs is balanced.

**Figure 5:**
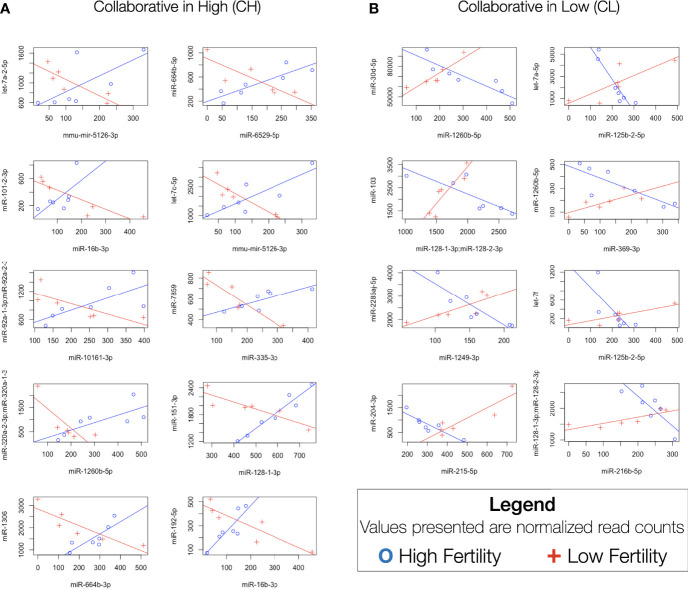
Significantly differentially correlated miRNAs. Difference in Spearman correlations between miRNA pairs in enhanced and standard fertility bulls. **(A)** CH pairs, positively correlated in high fertility animals and negatively correlated in low fertility. **(B)** CL pairs, positively correlated in low fertility animals, negatively correlated in high fertility.

In an attempt to more accurately model complex transcriptomic interactions in which miRNAs present in sperm may participate, a correlation-based analysis was performed to identify differences in miRNA-miRNA relationships. Analysis of 30381 unique two-way comparisons between miRNAs with robust expression revealed 18 miRNA pairs with significant Spearman correlations in both high and low fertility bulls. Our interpretation of the functional significance of these correlations is outlined in **Figure 4**. Of these pairs, 10 demonstrate “cooperative” characteristics in higher fertility bulls (CH): they are positively correlated in enhanced fertility bulls and negatively correlated in lower fertility individuals (**Figure 5A**). The remaining 8 pairs were “cooperative in lower fertility” (CL): negatively correlated in high fertility bulls and positively correlated in low fertility animals (**Figure 5B**).

#### 3.2.4 Target Analysis

Validated targeting interactions by both miRNAs of a correlated pair was reported for several genes for both CH (**Supplementary Table S3**) and CL (**Supplementary Table S4**) pairs. Target genes actively expressed in mature bovine testis (52), and presumed to be involved in spermatogenesis, are presented in **Supplementary Tables S5** and **S6**.

**Figure 6:**
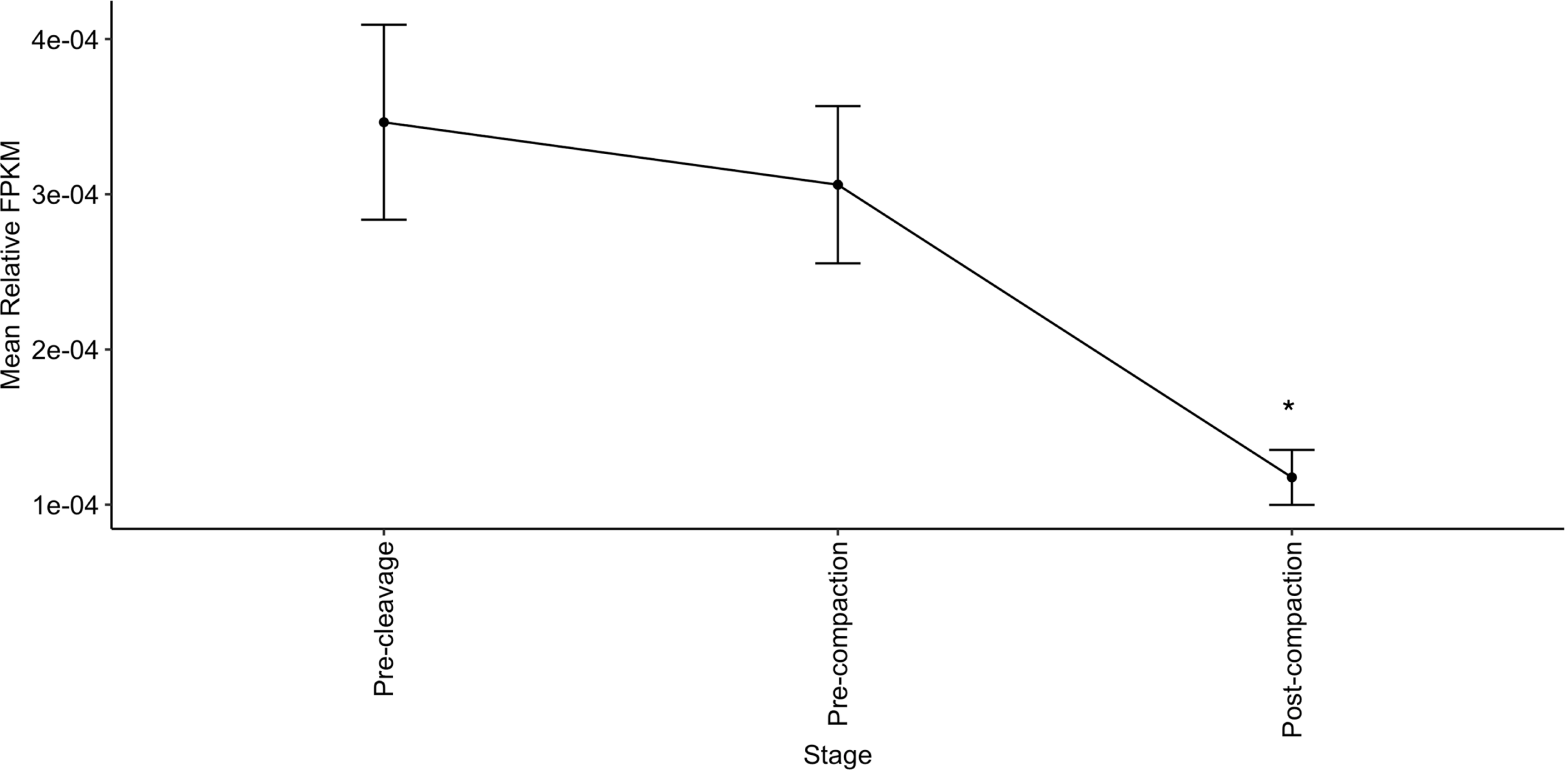
Relative expression of genes co-targeted by the most correlated miRNA pairs is highest in early embryonic stages. Relative abundance of genes targeted by both members of 3+ CH pairs or 4+ CL pairs declines in early embryos from pre-cleavage (GV, MII, 1-cell) and pre-compaction (2-cells, 4-cells, 8-cells-early, 8-cells-late) to post-compaction (morula, blastocyst-early, blastocyst-late). Expression data from EmbryoGENE Profiler (53). *indicates a significant difference in mean.

Targets expressed in the early embryo were similarly identified using the embryoGene database (53), shown in **Supplementary Tables S7** and **S8**. Expression of targets shared by both members of several CH or CL pairs is significantly lower post-compaction (**Figure 6**).

## 4 Discussion

### 4.1 Small Non-Coding RNA Population

In the present study, no statistically-significant differences in overall biotype composition of sncRNA populations were evident between higher and low fertility bulls. Each subtype of small RNA examined has been linked to one or more specific roles in essential sperm developmental stages or in conferring observable traits such as motility and morphology (14, 19, 54, 55) so this is not surprising. Large-scale sncRNA population changes would likely result in non-viable sperm, and samples from an animal demonstrating such a phenotype would be rapidly eliminated by quality control at the collection or management stage. Rather, differences are expected to be observed between a minor subset of influential sequences. Specific seminal tRNA fragments have been linked to *in vitro* success (56), while piRNA processing has been associated with creation of viable sperm (57), however, functionally relevant differential expression of individual sncRNA sequences was not observed in our model of idiopathic subfertility, so we elected to focus on further interrogating the miRNA population.

### 4.2 miRNAs

#### 4.2.1 Population Characteristics and Functional Analysis of Targets

**Figure 7:**
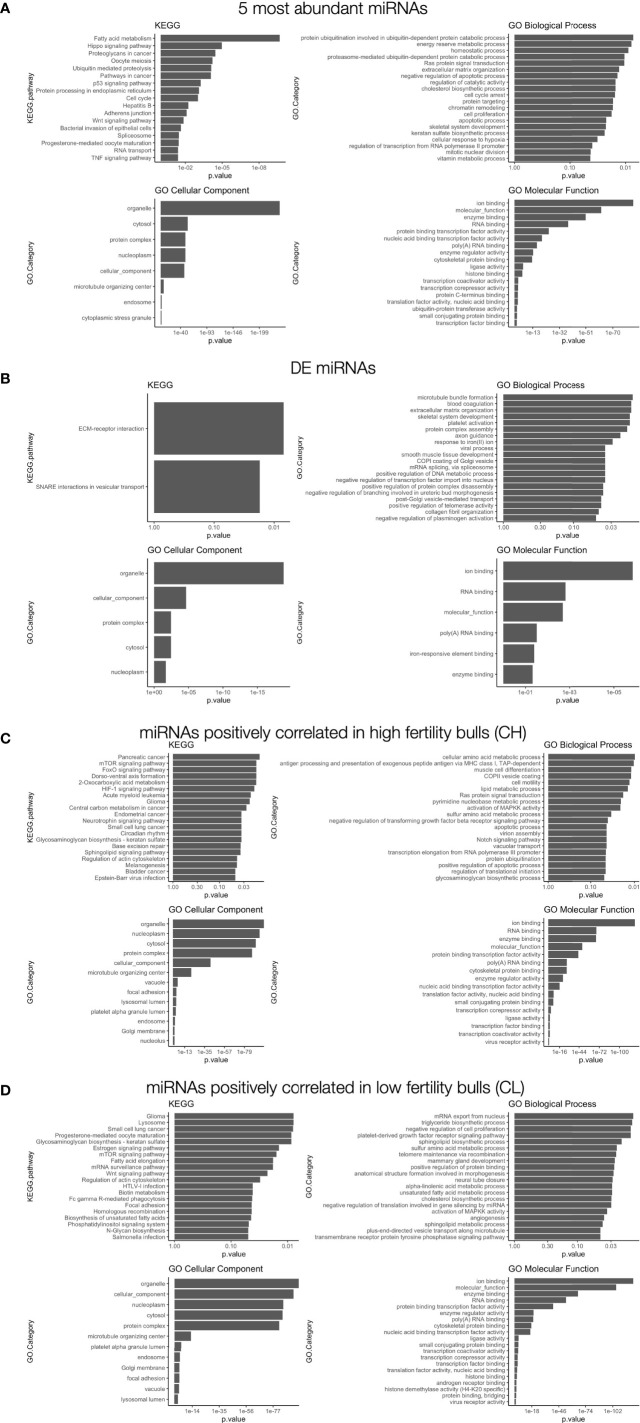
Gene set enrichment analysis of differentially correlated miRNAs. **(A)** GO and KEGG results for **(A)** the 5 most abundant miRNAs: miR-34b-3p; miR-100-5p; miR-191-5p; miR-30d-4p; miR-21-5p), **(B)** differentially expressed miRNAs with mean read count > 40: miR-409a-3p; miR-543), **(C)** differentially correlated sequences with positive correlations in enhanced fertility bulls, **(D)** differentially correlated sequences with positive correlations in standard fertility bulls. The top 20 most significantly enriched terms are shown.

miRNAs present in mature sperm are likely to reflect spermatogenic developmental or maturation processes (27, 28, 52). They may also represent functional post-transcriptional regulators with the potential to influence gene expression in the developing embryo (30, 31). No significant differences in the levels of these miRNA was evident between HF and LF bulls. Nevertheless, because miRNA abundance is highly relevant to the targeting and overall effect on gene expression in both contexts, we examined the most abundant miRNAs present to gain further insight into their potential roles in fertility. Validated target genes of the 5 most abundant miRNAs, representing 60% of all miRNA reads, were investigated by gene set enrichment analysis (KEGG and GO) to identify associated functional pathways (**Figure 7A**). The most significantly enriched KEGG term among the target RNAs was “fatty acid metabolism”. Regulation of membrane integrity is strongly linked to fertility (58), so the present findings suggest sperm-borne miRNAs have strong potential to participate in this pathway. By way of example, miR-191-5p and miR-30d target CLDND1 which shows increased expression in subfertile bulls (58). The reported interactions between these sequences and others (57), in light of the present findings, may suggest functional mechanisms by which these miRNAs influence membrane biology in developing sperm.

Several other abundant miRNAs from this study (and their targets) have been broadly linked to sperm viability, and many also play significant roles in female gametes. The miR-34 family is essential to spermatogenesis in bovine and mouse (14, 19). While other members are common in both spermatozoa and oocytes, miR-34b is less abundant in oocytes (19), and reduced expression in sperm is associated with reduced fertility in humans (59). Both miR-34b and miR-30d-5p are associated with sperm motility in Bos taurus (54). miR-21-5p is tightly linked to female gamete development and environmental responses (20, 60) and renewal of spermatogonial stem cells (61). All five miRNAs identified in this study have been previously reported in sperm from several species, including bovine, murine, and human (18, 62, 63), indicating conserved functional significance.

A small set of miRNAs were found to be differentially expressed with FDR < 0.05. These putative biomarkers exhibited higher expression in animals with higher fertility. Further investigation with greater sensitivity and an expanded sample size would be necessary to confirm the significance of these markers, due to their low levels of expression. Several of these sequences do not have human homologs available to allow thorough gene targeting analysis as performed for other homologous miRNAs. However, for 2 DE sequences that have human homologues, miR-409a-3p and miR-543, gene set enrichment investigation for specific targets was performed (**Figure 7B**). Six gene targets are shared by the two miRNAs: TNRC6A, MED10, CSDE1, FOXP1, MTA1, and SRP54. As stated in the results, based on the low levels of expression for all differentially-expressed miRNAs, no definitive statements can be made regarding their potential roles in fertility differences.

#### 4.2.2 IsomiR Expression

The results presented here suggest that differential expression of individual isomiRs within sperm samples does not appear to correlate with idiopathic subfertility in Holsteins. Interestingly, isomiR composition is known to vary between species and in response to different environmental stimuli (64). This suggests that the relatively consistent proportion of individual isomiRs present in the sperm samples examined, may be a result of the similarities in housing, management and collection procedures for high quality bulls. Our observation that the most highly expressed isomiRs for the most abundant miRNAs are distinct from the miRBase consensus sequence suggests that there may be testis-specific processing of miRNA sequences, though the implications of this remain unclear (**Figure 3**).

IsomiR variation occurs most commonly at the 3’ end, and does not typically involve variation in the seed sequence (nucleotides 2-8), which determines gene targeting (37). However, in some cases isomiRs sequences impact miRNA function (38). Specifically, 3’ editing may alter miRNA stability (65) or, rarely, targeting through 3’ supplementary interactions (66). Importantly 3’ sequence variation between isomiRs presents a technical challenge when attempting to validate next-generation sequencing results by RT-qPCR or RT-ddPCR, as specificity at the 3’ end is essential to the design of high quality primer sequences (67).

#### 4.2.3 Differential Correlation

The absence of significant patterns of differential miRNA expression between the two fertility groups led us to explore more complex associations in the miRNAome of our sequenced sperm samples that might be associated with fertility. Specifically, we interrogated our sequence data to identify specific correlated pairs of miRNAs with different relationships between the groups. Investigation of miRNA pairs in human sperm has been performed previously (39, 68), however the analysis in these studies focused on identifying pairs tightly correlated in fertile humans and uncorrelated in subfertile men (39, 63). This approach may be biased towards miRNAs for which expression is influenced by a common transcription factor, making the results more reflective of that specific regulatory factor, rather than the transcriptomic environment as a whole.

The novel approach taken in this study imposes more restrictive conditions for identifying significant correlations, as it requires an inverse trend to exist between fertility conditions. These conditions should ideally increase the specificity to capture important transcriptomic shifts and significant relationships between miRNAs that are correlated with specific miRNA functions and larger patterns of expression. Correlated miRNAs have previously been shown to regulate a single gene through synergistic interactions (69–71). The analysis described here identifies several cases where a cooperative relationship is observed in one fertility condition, while the opposite pattern of miRNAs in the other fertility condition, which suggests antagonistic effects at the level of target gene expression. For miRNAs that appear “cooperative,” a positive correlation between two miRNAs is associated with a given fertility condition, suggesting that both sequences likely function to maximally inhibit the expression of common RNA targets that influence fertility. The shared targets of the CH pairs are listed in **Supplementary Table S3** and include: TNPO1, MED13, SLC7A2, TM9SF3, PLAG2, and IL6ST. In contrast, pairs cooperative in low fertility animals are presented in **Supplementary Table S4** and include: UHMK1, APP, VCPIP1, TNP01, QKI, etc. It should be noted that the positively correlated “cooperative” relationships are only half of the observed trends. The negative correlations between paired miRNAs in the opposing fertility condition present a more complex and potentially interesting relationship from a functional perspective. If these correlated miRNAs do in fact cooperate functionally, they may act to selectively suppress specific gene expression, while permitting the expression of others to simultaneously regulate different components of a larger pathway. Alternatively, both miRNAs may target a single gene. In this case, the “net” activity of negatively correlated miRNAs would cooperatively regulate overall transcript abundance in a titratable manner. Dose-dependent interactions have been observed for several factors influencing female fertility (72, 73), and could contribute to optimal male fertility, either during spermatogenesis or within the newly-formed zygote. A model proposing the potential actions of such interactions is presented in **Figure 4**. Note that this model relies on “canonical” interactions of miRNAs downregulating genes, however non-canonical interactions such as upregulation are known to occur (74).

GO and KEGG analyses were performed to identify pathways implicated for CH (**Figure 7C**) or CL correlated miRNA pairs (**Figure 7D**). Notable pathways targeted by these pairs include cell motility in enhanced fertility samples, suggesting that some aspects of sperm transport may be altered between samples despite comparable macroscopic motility parameters. In addition, negative regulation of cell proliferation is a targeted process in CL pairs, suggesting that embryogenesis may be comparatively hindered in lower fertility bulls.

To explore these relationships further, we investigated several individual gene targets of both CH and CL pairs (**Supplementary Tables S3, S4**). Results were filtered to identify targets that are expressed in the testis (52) (**Supplementary Tables S5, S6**) or early embryo, up to the 8-cell stage (53) (**Supplementary Tables S7, S8**). Targets expressed in the testis are likely to interact with miRNAs during spermatogenesis to influence sperm development (75). Genes in the early embryo may be targeted by sperm-borne miRNAs to influence development (16, 30). To identify relevant potential target genes/transcripts we focused on embryos at the 8-cell stage and earlier which contain maternally-derived transcripts, since bovine embryonic genome activation begins at the 8-16 cell stage (76). Given that miRNAs typically degrade within a few days, these sequences are the most likely to act as targets for any sperm-borne RNAs present.

*In silico* analysis of our miRNAs against a report of bovine testis-expressed genes (52), revealed that predicted target transcripts of CH miRNAs are expressed in the testis (**Supplementary Table S5**). These include RHOA (involved in capacitation and the acrosome reaction) (77) and Cdc7, associated with arrested DNA synthesis during spermatogenesis (78). Using Cdc7 as an example gene in our proposed model of correlated miRNA function, cooperative miRNA interactions in a CH model would selectively permit or suppress Cdc7 expression as appropriate for the stage of spermatogenesis, effectively regulating the DNA damage checkpoint. In contrast, if the pair is regulated in a direction that results in antagonistic miRNA expression, only partial suppression would be expected, theoretically allowing continued development of sperm with higher levels of DNA damage and lower fertility.

A second potential site of action of sperm-borne miRNAs is in the zygote following fertilization. Rapid changes in gene expression, including the decay of maternal transcripts, are known to occur at this time, and small RNAs have been implicated in this process (41, 79, 80). As identified in **Figure 6**, genes targeted by several of the implicated miRNAs exhibit their highest relative expression in the pre-compaction stages of embryogenesis. High expression is thought to reflect their importance in early development, making these potentially interesting functional targets of the miRNA pairs. We therefore interrogated the EmbryoGENE (53) database for CH target sequences expressed prior to the 8-cell embryo stage. Several validated CH targets are expressed in the early embryo, such as: TNPO1, which is linked to bovine fertility (81) and equine pregnancy recognition (82); MED13, which is linked to zygotic genome activation and post-implantation development (83); and the murine Plag1 family (to which PLAGL2 belongs), which is implicated in timing of zygotic genome activation (84). Differences in miRNA-dependent targeting of these post-fertilization processes could contribute to subtle fertility differences, by altering the timing and extent of gene expression in this critical developmental window. These miRNAs may work in parallel to endocrine factors regulating early development, suppressing genes until their optimal period of expression. For example: TNPO1 is linked to regulation of estrogen receptor-α (85), IL6ST is regulated by progesterone and interferon tau during early pregnancy (86), and PLAG2 proteins are activated in the placenta in response to TGFα and leptin (87). We postulate that sperm-borne RNAs may cooperate to regulate these processes, facilitating precise, temporally-appropriate, alterations in key developmental steps. A complete list of CH pair targets expressed in the embryo is presented in **Supplementary Table S7**.

Target genes of miRNA pairs correlated in lower fertility bulls (CL) and expressed in the testis (**Supplementary Table S6**) include the PKD1 family, which has been associated with sperm movement in the female tract (88), and has a sperm-specific receptor expressed in humans (89). Also present is ID2, which is implicated in progression through pachytene during spermatogenic meiosis (90), and suppression of ID2 causes spermatogenesis failure in mice (91). Embryo-expressed targets include LARP1, a poly-A binding protein shown to be essential for male fertility and early embryogenesis in a Drosophila model (92) and MAP1B, for which SNPs have been implicated in altered SCR and fertilization rates *in vitro* (11). Our findings predict that miRNA-mediated MAP1B suppression may also impact fertility. Another target of CL miRNA pairs, MATR3, is known to be more abundant in males with low motility (93). Because gross assessment of motility appears comparable between the lower and enhanced fertility samples used in this study, it is possible that miRNAs contribute to MATR3-dependent motility characteristics which are not apparent in standard approaches, but which still impact fertility. These and many other target genes of the miRNA correlated pairs described are expressed in both sperm and early embryos, so it is plausible that the correlated pairs actually regulate gene expression in both contexts.

The overall goal of the present study was to characterize miRNA populations present in bovine sperm samples that were normal with respect to standard metrics of quality (motility, morphology etc.), but varied with respect to fertility. Previous work investigating differentially expressed miRNAs linked to motility implicated few members of the correlated pairs identified here (let-7a, let-7c, and miR-103), which were more highly expressed in the high motility fraction, while the rest showed no association (54). The absence of such differences in the present work was expected, as bulls with sperm showing abnormal motility are typically eliminated from potential breeding stock. This work has identified the most prevalent miRNAs in the sperm of Holstein bulls and characterized molecular variation (isomiRs) and validated targets of those miRNAs that are potentially relevant to spermatogenesis, fertilization and early embryo development. Importantly, correlations involving specific miRNAs were identified that may therefore be reflective of specific interactions in the control of gene expression related to bull fertility. This observation of correlation-based associations may reflect more subtle differences in sperm transcriptome the dynamics of the sperm transcriptome and provides an opportunity for a more detailed investigation into the contribution of miRNAs to fertility.

## Data Availability Statement

The original contributions presented in the study are publicly available. This data can be found here: BioProject, PRJNA777266.

## Author Contributions

Conceptualization, NW, SR, CL, SM, and JL; methodology, NW, SR, and JL; formal analysis, NW, SR, and DG; investigation, NW; resources, KH, SM, SL, and ML; data curation, NW and SR; writing—original draft preparation, NW; writing—review and editing, NW, SR, SM, CL, and JL; visualization, NW; supervision, JL and CL; funding acquisition, JL and CL. All authors have read and agreed to the published version of the manuscript.

## Funding

This research was funded by the Natural Sciences and Engineering Research Council (NSERC Discovery: RGPIN 04396) (JL) and the Ontario Ministry of Agriculture and Food (OMAFRA: UG-T1-2020-100265) (JL). NW is the recipient of an OMAFRA HQP Scholarship and a Scholarship from the Ontario Veterinary College.

## Conflict of Interest

The authors declare that the research was conducted in the absence of any commercial or financial relationships that could be construed as a potential conflict of interest.

## Publisher’s Note

All claims expressed in this article are solely those of the authors and do not necessarily represent those of their affiliated organizations, or those of the publisher, the editors and the reviewers. Any product that may be evaluated in this article, or claim that may be made by its manufacturer, is not guaranteed or endorsed by the publisher.
